# A Genome-Wide Association Study Identifies *UGT1A1* as a Regulator of Serum Cell-Free DNA in Young Adults: The Cardiovascular Risk in Young Finns Study

**DOI:** 10.1371/journal.pone.0035426

**Published:** 2012-04-12

**Authors:** Juulia Jylhävä, Leo-Pekka Lyytikäinen, Mika Kähönen, Nina Hutri-Kähönen, Johannes Kettunen, Jorma Viikari, Olli T. Raitakari, Terho Lehtimäki, Mikko Hurme

**Affiliations:** 1 Department of Microbiology and Immunology, School of Medicine, University of Tampere, Tampere, Finland; 2 Department of Clinical Chemistry, School of Medicine, University of Tampere, Tampere, Finland; 3 Department of Clinical Physiology, University of Tampere and Tampere University Hospital, Tampere, Finland; 4 Department of Pediatrics, University of Tampere and Tampere University Hospital, Tampere, Finland; 5 Institute for Molecular Medicine Finland (FIMM), University of Helsinki, Helsinki, Finland; 6 Department of Medicine, University of Turku and Turku University Hospital, Turku, Finland; 7 Research Centre of Applied and Preventive Cardiovascular Medicine, University of Turku, Turku, Finland; 8 Department of Clinical Physiology, University of Turku and Turku University Hospital, Turku, Finland; 9 Department of Clinical Chemistry, Tampere University Hospital, Tampere, Finland; 10 Department of Microbiology, Tampere University Hospital, Tampere, Finland; Universite de Montreal, Canada

## Abstract

**Introduction:**

Circulating cell-free DNA (cf-DNA) is a useful indicator of cell death, and it can also be used to predict outcomes in various clinical disorders. Several innate immune mechanisms are known to be involved in eliminating DNA and chromatin-related material as part of the inhibition of potentially harmful autoimmune responses. However, the exact molecular mechanism underlying the clearance of circulating cf-DNA is currently unclear.

**Methods:**

To examine the mechanisms controlling serum levels of cf-DNA, we carried out a genome-wide association analysis (GWA) in a cohort of young adults (aged 24–39 years; n = 1841; 1018 women and 823 men) participating in the Cardiovascular Risk in Young Finns Study. Genotyping was performed with a custom-built Illumina Human 670 k BeadChip. The Quant-iT^TM^ high sensitivity DNA assay was used to measure cf-DNA directly from serum.

**Results:**

The results revealed that 110 single nucleotide polymorphisms (SNPs) were associated with serum cf-DNA with genome-wide significance (p<5×10^−8^). All of these significant SNPs were localised to chromosome 2q37, near the *UDP-glucuronosyltransferase 1 (UGT1)* family locus, and the most significant SNPs localised within the *UGT1 polypeptide A1* (*UGT1A1*) gene region.

**Conclusion:**

The UGT1A1 enzyme catalyses the detoxification of several drugs and the turnover of many xenobiotic and endogenous compounds by glucuronidating its substrates. These data indicate that UGT1A1-associated processes are also involved in the regulation of serum cf-DNA concentrations.

## Introduction

Circulating cell-free DNA (cf-DNA) was first described in 1940s [1], and it has frequently been used as a convenient indicator of cell death and tissue damage in various acute and chronic disorders [2]–[4]. Detectable amounts of cf-DNA are present in healthy individuals, yet significantly increased plasma/serum levels of cf-DNA have been reported in patients with acute cardiovascular disease, sepsis, cancer and pre-eclampsia [2]–[4]. Circulating nucleosomal DNA has also been proposed as a major autoantigen in systemic lupus erythematosus [5] and as a potential player in lupus nephritis [6]. Additionally, genomic DNA fragments released during sterile thyroid injury may also trigger autoreactivity-related thyroid dysfunction [7]. Other potentially detrimental functions implicated for cf-DNA include the horizontal transfer of oncogenic sequences (i.e. genometastasis) [8] and the creation of a proinflammatory milieu in the circulation [9]. Endogenous or microbial DNA are also known to act as alerting signals to the host immune system, and various soluble, membrane-bound and intracellular receptors that can recognise DNA have been identified. These receptors include pattern recognition molecules such as collectins, ficolins, pentraxins, soluble CD14 and C1q [10]; Toll-like receptors (TLRs) [11]; the high-mobility group box (HMGB) proteins [12]; components of the inflammasome [13]; and extrachromosomal histone H2B [7]. Upon sensing DNA, these receptors can mount an immuno-inflammatory response to eliminate the circulating DNA and prevent anti-DNA autoimmunity.

Despite its proven utility in diagnosis and in determining various clinical conditions, many aspects ofthe origin, metabolism and non-pathological fluctuations of cf-DNA remain unresolved. According to the current understanding, the majority of cf-DNA is derived from apoptotic or necrotic cells; however, active secretion by leukocytes may also contribute to the pool of circulatory DNA [4]. Serum cf-DNA is hypothesised to consist of both free DNA and particle-bound forms [4], yet the relationships between cf-DNA composition, originating cell types and the given clinical conditions are unclear.

Studies in mice have demonstrated that injected DNA, including single-stranded DNA (ssDNA), double-stranded DNA (dsDNA), nucleosomal core particles and oligonucleotides, is cleared from the system very rapidly, in less than 40 minutes, and that the major organ responsible for the solubilisation and generation of DNA degradation products is the liver [14]–[16]. Nevertheless, the clearance kinetics of these distinct DNA speciesare somewhat different, and the induction of the acute phase response or co-injection of the acute phase reactants serum amyloid P (SAP) and C-reactive protein (CRP) causes a delay in chromatin clearance, concomitant with an increased localisation of cf-DNA to the liver [17]. However, it has also been demonstrated that macrophages play an essential role in processing the cf-DNA released from dead or dying cells [18], [19]. Data regarding the corresponding cf-DNA dynamics in humans are very scarce, but it has been reported that foetal cf-DNA has a mean half-life of 16.3 minutes in the maternal plasma and that plasma nucleases have only a partial role in the removal of foetal DNA [20].

To identify the genetic determinants of serum cf-DNA levels and thus elucidate the mechanisms involved in cf-DNA turnover, we performed a genome-wide association analysis (GWA) in a cohort of young adults participating in the Cardiovascular Risk of Young Finns study.

## Results

**Figure 1:**
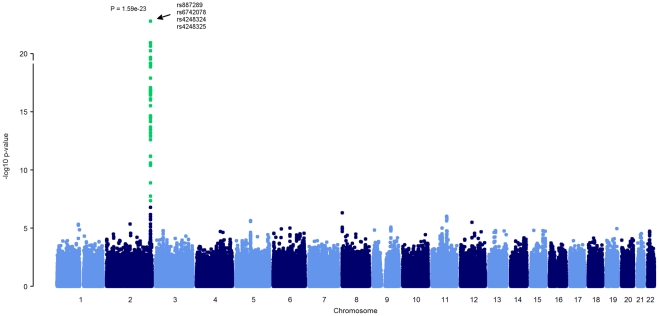
Manhattan plot of the genome-wide analysis of SNPs associated with serum cf-DNA levels. SNPs with significant genome-wide p-values (p<5×10^−8^) are indicated in green.

**Figure 2:**
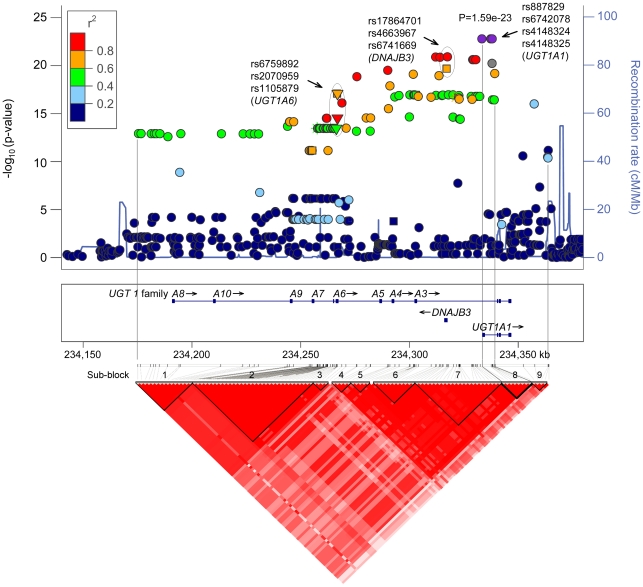
*UGT1* regional association plot and haploblock structure of the genome-wide analysis of SNPs associated with serum cf-DNA levels. The violet circles indicate the top SNPs in the *UGT1A1* region, the circled triangles indicate the non-synonymous SNPs in *UGT1A6* region, and the circled squares and circles indicate the SNPs in the *DNAJ3B* promoter region. The colour of the spots indicates LD (data from HapMap II CEU) with the index SNPs (violet spots). The blue line shows the recombination rate across the region (data from HapMap II CEU). The nine sub-blocks in the major haploblock are separated with black lines. The sub-block containing the four top SNPs is separated with a thick black line.

The characteristics of the study population are presented in Table 1. In addition to age and insulin concentration, all of the variables tested differed significantly between women and men (Table 1). In the GWA, a total of 110 single nucleotide polymorphisms (SNPs) were associated with serum cf-DNA levels with a genome wide significance level of p<5×10^−8^. All 110 SNPs localised to the *UDP-glucuronosyltransferase 1 (UGT1)* family locus on chromosome 2q37, and the most significant SNPs resided in the *UGT1 polypeptide A1* (*UGT1A1)* gene region (Figure 1 and Figure 2). In the *UGT1* gene family region, we observed a major haploblock that could be further divided into nine sub-blocks (using the four gamete rule in HaploView). The four top SNPs on *UGT1A1* belong to the sub-block no. 8, which spans approximately 11 kb, and these SNPs appeared to be in virtually perfect linkage (*r^2^ = 0.9991−1.0*). None of the significant SNPs in the sub-block no. 8 resided in exonic regions. However, among the significant SNPs outside this haploblock, three non-synonymous SNPs (rs6759892, rs2070959, rs1105879, p = 8.2×10^−18^−3.4×10^−14^) were identified in the coding region of *UGT1A6* (Figure 2). In addition, three significant SNPs (rs17864701, rs4663967, rs6741669, p = 1.2×10^−21^−1.1×10^−17^) were detected in the promoter region of *DNAJB3* (Figure 2). The top SNPs in each of the nine sub-blocks are presented in Table 2.

**Table 1:** Characteristics of the study population.

Variable	Women (n = 1018)		Men (n = 823)		p for difference
	Mean	S.D.	Mean	S.D.	
Age (years)	31.7	5.0	31.7	5.0	N.S.
BMI (kg/m^2^)	24.4	4.5	25.7	4.2	<0.001
Waist circumference (cm)	79.2	11.2	90.0	11.0	<0.001
Systolic blood pressure (mmHg)	113	12	121	13	<0.001
Diastolic blood pressure (mmHg)	69	10	73	11	<0.001
Total cholesterol (mmol/L)	5.02	0.87	5.20	1.00	<0.001
HDL-cholesterol (mmol/L)	1.39	0.29	1.16	0.28	<0.001
LDL-cholesterol (mmol/L)	3.11	0.76	3.40	0.89	<0.001
Triglycerides (mmol/L)*	1.0	0.8–1.3	1.2	0.9–1.8	<0.001
Insulin (mU/L)*	6	5–9	6	4–9	N.S.
Glucose (mmol/L)*	4.9	4.6–5.1	5.1	4.9–5.4	<0.001
Homocysteine (μmol/L)*	8.5	7.2–10.1	10.1	8.7–11.7	<0.001
cf-DNA (μg/ml)	0.755	0.135	0.862	0.111	<0.001
CRP (mg/L)*	0.84	0.35–2.30	0.59	0.30–1.42	<0.001
Alcohol (drinks per week)*	2	0–5	6	1–13	<0.001
Smoking daily (% of total)†	19.3		29.4		<0.001
Use of COCs (% of total)	24.9				

* Median values and interquartile range (IQR), Mann-Whitney test for between-group differences.

† Chi-squared test for differences between groups.

Abbreviations: BMI,body mass index; cf-DNA, cell-free DNA; COCs, combined oral contraceptives; CRP, C-reactive protein; N.S., not significant.

**Table 2:** The top SNPs in each of the 9 sub-blocks in the *UGT1A* gene region (see Figure 2).

SNP†	Sub-block	Locus (bp)	Ancestral allele	Minor allele	MAF	β (S.E.)	p*	Imputed	Location
rs17864683	1	234243948	A	C	0.3221	0.3246 (0.0352)	2.06E−14	yes	intron, UGT1A10
rs10168416	2	234261826	G	C	0.4230	0.3236 (0.0325)	2.98E−15	no	intron, UGT1A8
rs1105879	3	234266941	C	A	0.4603	0.3127 (0.0324)	8.20E−18	no	missense, UGT1A6
rs17863787	4	234275833	G	T	0.3592	0.3092 (0.0338)	1.40E-19	no	intron, UGT1A6
rs6744284	5	234290036	T	C	0.3710	0.2818 (0.0335)	3.04E−20	no	intron, UGT1A6
rs6722076	6	234312056	A	G	0.3705	0.2824 (0.0334)	1.25E−21	yes	intron, UGT1A6
rs17864701	7	234317456	T	C	0.3722	0.2591 (0.0335)	1.19E−21	yes	intron, UGT1A6
rs887829	8	234333309	A	G	0.3983	0.3358 (0.0331)	1.59E−23	no	intron, UGT1A1
rs6742078		234337378	T	G	0.3983	0.3358 (0.0331)	1.59E−23	no	intron, UGT1A1
rs4148324		234337461	G	T	0.3983	0.3358 (0.0331)	1.59E−23	no	intron, UGT1A1
rs4148325		234338048	T	C	0.3983	0.3358 (0.0331)	1.59E−23	no	intron, UGT1A1
rs11690786	9	234357356	T	C	0.3839	0.2711 (0.0337)	9.67E−17	yes	intron, HEATR7B1

The four SNPs that are associated most significantly with serum cf-DNA levels are in sub-block 8.

† For each SNP, the ancestral allele was modelled, and the β coefficient represents the change in cf-DNA level (µg/ml) with one additional copy of the ancestral allele.

* Adjusted for age, sex, BMI and genetic East-West stratification in the Finnish population.

Abbreviations: SNP, single nucleotide polymorphism; Chr, chromosome; bp, base pairs; MAF, minor allele frequency; S.E., standard error.

When the selected tag SNPs from each sub-block were analysed using the stepwise AIC model selection algorithm, only the tag SNP rs4148324 which identifies the sub-haploblock no. 8 containing the four top SNPs, remained in the model. This SNP was found to explain 5.3% of the total variation in serum cf-DNA levels. In addition, conditioning the analysis on the top SNP rs4148324 (i.e. additional adjustment of the model with the top SNP) did not reveal any other SNPs associated with cf-DNA levels at a genome-wide significance level (all p-values>0.4).

**Figure 3:**
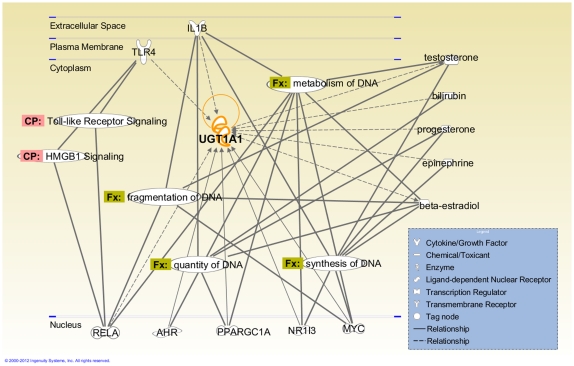
The UGT1A1 interaction network generated using the Ingenuity Pathway Analysis. The molecular relationships connecting UGT1A1 to DNA metabolism synthesis, fragmentation and quantity of DNA are indicated with Fx symbols, and the associated Canonical Pathways are indicated with CP symbols. Abbreviations: AHR, aryl hydrocarbon receptor; HMGB1, high-mobility group box protein 1; IL1B, interleukin-1 beta; MYC, v-myc myelocytomatosis viral oncogene homolog; NR1I3, nuclear receptor subfamily 1, group I, member 3; PPARGC1A, peroxisome proliferator –activated receptor gamma coactivator 1 alpha; RELA, v-rel reticuloendotheliosis viral oncogene homolog A (avian); TLR4, Toll-like receptor 4.

An interaction network for UGT1A1 constructed with IPA (Ingenuity Pathway Analysis) revealed that several molecules in the UGT1A1 interaction network are connected to DNA metabolism, quantity, fragmentation and synthesis of DNA (Figure 3). In addition, molecular connections to HMGB signalling and TLR signalling were observed for UGT1A1 in the IPA Canonical Pathway analysis (Figure 3)

## Discussion

The circulating cf-DNA concentration has been shown to increase during pathological processes, reflecting the rate of cellular death and tissue damage. However, the regulation and metabolism of cf-DNA have received much less attention, and data regarding the turnover of cf-DNA are scarce. In this study, we demonstrate that a novel candidate gene, UGT1A1, may contribute to the regulation of serum cf-DNA in young adults. The UGTs comprise a family of enzymes that primarily transfer glucuronic acid to hydroxyl, carboxylic acid and amine group-bearing compounds to facilitate the catabolism and clearance of these compounds [21]. Because a wide variety of compounds contain these groups, glucuronidation provides a common mechanism for the elimination of several endobiotics, xenobiotics and drugs. The *UGT1A* locus encodes several isoforms of the enzyme, each with a tissue-specific expression pattern [21]. Although the liver is the major producer of UGT1A1, the expression of this isoform has also been detected in the bile duct, the stomach, the small intestine, the kidneys, the bladder, the uterus and the colon [21], [22]. Interestingly, the expression of UGT1A1 has recently been demonstrated in human peripheral blood mononuclear cells [23].

Due to the strong linkage disequilibrium between the SNPs in the *UGT1* region (Figure 2) we cannot completely rule out the involvement of the other significant SNPs, such as those in the coding region of *UGT1A6* or in the promoter of *DNAJB3*, in the regulation of cf-DNA levels. Nevertheless, the main signal can be confined to the sub-block of the four top *UGT1A1* SNPs because the significance of the p-values of the other SNPs decreases along with the increase in distance from this sub-block (Table 2 and Figure 2).

One of the best characterised functions of UGT1A1 is the glucuronidation of bilirubin [21], and numerous recent GWA studies have identified *UGT1A1* alleles as the major regulators of serum bilirubin levels [24]. The four most significant SNPs associated with serum cf-DNA levels in this study (rs4148324, rs6742078, rs4148325 and rs887829) have also been identified as regulators of serum bilirubin concentrations [24]. The effect of these SNPs on bilirubin levels is, however, reported to be due to a functional TA-repeat polymorphism in the *UGT1A1* promoter TATA-box area; recent conditional linkage scans of this TA-repeat association demonstrated that it accounts for the linkage peaks observed for the other associated loci in the gene region 25,26. Given that the ancestral alleles of the rs4148324, rs6742078, rs4148325 and rs887829 SNPs are in strong linkage with the wild-type form of the TA-repeat polymorphism (TA_6,_ rs8175347), and because all of these variants belong to the same haploblock in our population, we assume that the same regulation pattern holds true for both serum cf-DNA and bilirubin levels. The TA_6_ form of the repeat polymorphism is associated with lower bilirubin levels due to higher promoter activity (via optimal transcription factor IID binding), resulting in higher UGT1A1 enzyme levels and enhanced bilirubin glucuronidation and clearance [27]. Because we observed lower cf-DNA levels in subjects bearing the wild type alleles (ß = −0.3358) of the four top SNPs in comparison with the minor allele carriers, we suggest that higher UGT1A1 production, or some cellular process associated with UGT1A1 induction, contributes to more effective turnover of serum cf-DNA.

Animal studies have demonstrated that the liver is the major site of cf-DNA clearance, although some portion of the circulating DNA also localises to other organs, such as the kidneys and spleen [14], [16], [17]. However, in humans, foetal cf-DNA has been shown to be cleared rapidly from the maternal circulation, and plasma nucleases have only a partial effect on cf-DNA disposal [20]. Moreover, relatively large DNA fragments (>150 base pairs) originating from non-renal or urinary tract tissues have been detected in the urine [28], indicating that the complete digestion of cf-DNA by nucleases is not required for its excretion. Beyond these observations, the metabolism and fates of different cf-DNA forms are unknown. Although our data does not provide a mechanistic basis of UGT1A1-mediated cf-DNA clearance, we propose that the metabolism of cf-DNA may be facilitated by UGT1A1, either directly by glucuronidation or indirectly via the interactions between UGT1A1 and the molecules related to DNA sensing and processing (Figure 3). It is also possible that a portion of the cf-DNA is endocytosed and/or co-processed with the traditional UGT1A1 substrates, such as the bilirubin-albumin complex [29].

Nevertheless, physiological interactions between UGT1A1 and nucleotides have been demonstrated [30] and the glucuronidation of a 2′,3′-dideoxynucleoside compound, the 3′-Azido-3′-deoxythymidine (AZT), has been demonstrated, although this reaction is performed by the UGT2B7 enzyme [31]. However, at the present time, we can only speculate whether these observations are related to the glucuronidation of endogenous cf-DNA during the course of its cellular internalisation or (apoptotic) release. Neither can we exclude the possibility that the actual UGT1A1 substrate might be some lipophilic or proteinaceous structure associated with cf-DNA. Furthermore, the potential involvement of extrahepatic UGT1A1-expression sites, such as leukocytes [23] or intestinal cells [32], in cf-DNA turnover cannot be addressed by our data.

Our statistical model for genetic variation in the UGTA1 explains only 5.3 % of the total variation in cf-DNA levels, although the association is highly statistically significant (p = 1.6×10^−23^). The somewhat modest coefficient of determination can be explained by the complex nature of the serum cf-DNA; different forms of cf-DNA might be assigned to different clearance pathways with dissimilar kinetics and saturability. It is also known that the serum contains higher cf-DNA levels than the plasma, allegedly due to the release of genomic DNA from leukocytes during blood clotting [33] Recently, however, this view has been challenged by the observation that leukocyte rupture is not the major factor causing increased serum cf-DNA levels [34]. Nevertheless, the strong association suggests a significant biological role for UGT1A1 in regulating cf-DNA levels, especially because no other polymorphisms outside the reported UGT1A1 loci emerged with genome-wide significance when the analysis was adjusted using the top SNP (rs4148324).

In conclusion, the results of this GWA study demonstrate that *UGT1A1* polymorphisms are associated with serum cf-DNA levels in young Finns. We propose that UGT1A1-associated processes are, either directly or indirectly, involved in the regulation of cf-DNA concentration. These results, however, are limited to one study cohort which is a major limitation of our study. Therefore the results should be replicated in an independent study cohort, and further investigation to establish the exact role of UGT1A1 in cf-DNA turnover is warranted.

## Materials and Methods

### Study population

The study population consisted of 1841participants in the Cardiovascular Risk in Young Finns study (aged 24–39 years; 1018 women and 823 men). The study was approved by the local ethics committees (the University Hospitals of Helsinki, Turku, Tampere, Kuopio and Oulu) and was conducted following the guidelines of the Declaration of Helsinki. All participants gave their written informed consent. For a detailed cohort description and the assessment of the variables listed in Table 1, see [35] and the references therein. The data in this study were from individuals with successful cf-DNA measurements and genotyping and who participated in the follow-up study in 2001.

### Quantification of serum cf-DNA

Total circulating cf-DNA was determined directly in previously unthawed serum using the fluorescence-based Quant-iT™ high-sensitivity DNA assay kit and a Qubit^®^ fluorometer (Invitrogen, Carlsbad, CA, USA). All samples were analysed in duplicate, and the mean of the two measurements was used as the final value. At the mean serum cf-DNA levels of 0.650 µg/ml, the assessed intra- and inter-day variation coefficients were 2.2% and 4.7%, respectively. At the mean serum cf-DNA level of 1.02 µg/ml, the intra- and inter-day variation coefficients were 3.0% and 5.8%, respectively.

### Genotyping

Genotyping was performed at the Welcome Trust Sanger Institute using a custom made Illumina Human 670 k BeadChips. Genotypes were determined using the Illumina clustering algorithm [36]. Fifty-sixsamples failed the Sanger genotyping pipeline QC criteria (i.e., duplicated samples, heterozygosity, low call rate, or Sequenom fingerprint discrepancies). Three samples were removed due to a low genotyping call rate (<0.95) and 54 samples were excluded for possible relatedness (pi.hat>0.2). A total of 11,766 single SNPs were excluded due to deviation from Hardy-Weinberg equilibrium (HWE) test (p≤1e-06), 7,746 SNPs failed the missingness test (call rate<0.95) and 34,596 SNPs failed the frequency test (MAF<0.01). After quality control, 546,677 genotyped SNPs remained available for further analysis. Genotype imputation was performed using MACH 1.0 [37], [38] and HapMap II CEU (release 22) samples as reference. After imputation, 2,543,887 imputed SNPs were available. SNPs with squared correlations ≥0.3 between imputed and true genotypes were considered well imputed.

### Statistical methods

The comparison of the basic study population variables presented in Table 1 was conducted with the Student's t-test, Mann-Whitney's test or chi-squared test. Prior to the GWA, all continuous variables were subjected to inverse normal transformation to minimise the incidence of type I errors and to reduce the impact of outliers [39]. A stepwise backward model (Akaike information criterion, AIC) was used to determine which covariates explained the most of the variation in the cf-DNA levels. In addition to the variables listed in Table 1, genetic main identity-by-descent (IBD) clustering components and interactions between age and gender as well as smoking status and the use of alcohol were included in the model. The variables that remained in the model and were thus used as the adjustment covariates in GWA were gender, age, systolic blood pressure, fasting glucose, triglycerides, CRP, homocysteine, daily smoking, use of alcohol, use of combined oral contraceptives, main IBD components and the gender*age and daily smoking*use of alcohol the interaction terms. These factors were observed to account for 30.5% of the variation in cf-DNA levels. To reveal other SNPs potentially associated with serum cf-DNA, the analysis was additionally adjusted with the top SNP, rs4148324. Standardised residuals were extracted from the model, and association analysis was performed using linear regression with an assumption of an additive genetic effect. PLINK [40] was used for the true genotyped SNPs, and ProbABEL [41] was used for imputed genotype dosages. If the same SNP was available in both genotyped and imputed form, the genotyped form was displayed and included in the results. A commonly accepted threshold for genome-wide statistical significance level (p<5×10^−8^) was used to identify significant SNPs. Manhattan and Q-Q plots were drawn to confirm the validity of the analysis. The genomic inflation factor (lambda) was 0.9996475. The variance in serum cf-DNA level explained by each SNP (R^2^) was calculated as follows: R^2^ = 1/[1+(number of samples−2)×(SE of SNP/beta estimate of SNP)^2^]. The individual genotype data for genome-wide significant SNPs in the same region were extracted from both the genotyped and imputed data; then the allele dosages of the imputed SNPs were rounded to best guess genotypes and transferred to Haploview [42] for haploblock analysis and tag SNP identification. Haploblocks were defined using the four gamete rule in Haploview, and the SNP with the lowest p-value in each haploblock was selected as the tag SNP. All tag SNPs were analysed with stepwise backward AIC to identify the potential independent signals in different haploblocks. Unless otherwise noted, the statistical analyses were performed the appropriate packages in R (MASS for stepwise AIC).

To identify the potential interactions via which UGT1A1 could be connected to cf-DNA regulation, a graph of the UGT1A1 molecular network and associated functions and Canonical Pathways was generated using IPA (Ingenuity® Systems, www.ingenuity.com). In the IPA network analysis, the biological relationship between two molecules is represented as an edge (continuous or dashed line). All edges are supported by at least one reference from the literature, textbook or canonical information stored in the Ingenuity Knowledge Base.

## References

[1] Mandel P, Metais P (1948). [Not Available].. C R Seances Soc Biol Fil.

[2] Tong YK, Lo YM (2006). Diagnostic developments involving cell-free (circulating) nucleic acids.. Clin Chim Acta.

[3] Tsang JC, Lo YM (2007). Circulating nucleic acids in plasma/serum.. Pathology.

[4] Peters DL, Pretorius PJ (2011). Origin, translocation and destination of extracellular occurring DNA - A new paradigm in genetic behaviour.. Clin Chim Acta.

[5] Decker P, Singh-Jasuja H, Haager S, Kotter I, Rammensee HG (2005). Nucleosome, the main autoantigen in systemic lupus erythematosus, induces direct dendritic cell activation via a MyD88-independent pathway: consequences on inflammation.. J Immunol.

[6] Fenton KA, Rekvig OP (2007). A central role of nucleosomes in lupus nephritis.. Ann N Y Acad Sci.

[7] Kawashima A, Tanigawa K, Akama T, Wu H, Sue M (2011). Fragments of genomic DNA released by injured cells activate innate immunity and suppress endocrine function in the thyroid.. Endocrinology.

[8] Garcia-Olmo D, Garcia-Olmo DC (2001). Functionality of circulating DNA: the hypothesis of genometastasis.. Ann N Y Acad Sci.

[9] Atamaniuk J, Kopecky C, Skoupy S, Saemann MD, Weichhart T (2011). Apoptotic cell-free DNA promotes inflammation in haemodialysis patients..

[10] Litvack ML, Palaniyar N (2010). Review: Soluble innate immune pattern-recognition proteins for clearing dying cells and cellular components: implications on exacerbating or resolving inflammation.. Innate Immun.

[11] Hornung V, Latz E (2010). Intracellular DNA recognition.. Nat Rev Immunol.

[12] Yanai H, Ban T, Wang Z, Choi MK, Kawamura T (2009). HMGB proteins function as universal sentinels for nucleic-acid-mediated innate immune responses.. Nature.

[13] Muruve DA, Petrilli V, Zaiss AK, White LR, Clark SA (2008). The inflammasome recognizes cytosolic microbial and host DNA and triggers an innate immune response.. Nature.

[14] Du Clos TW, Volzer MA, Hahn FF, Xiao R, Mold C (1999). Chromatin clearance in C57Bl/10 mice: interaction with heparan sulphate proteoglycans and receptors on Kupffer cells.. Clin Exp Immunol.

[15] Emlen W, Mannik M (1984). Effect of DNA size and strandedness on the in vivo clearance and organ localization of DNA.. Clin Exp Immunol.

[16] Gauthier VJ, Tyler LN, Mannik M (1996). Blood clearance kinetics and liver uptake of mononucleosomes in mice.. J Immunol.

[17] Burlingame RW, Volzer MA, Harris J, Du Clos TW (1996). The effect of acute phase proteins on clearance of chromatin from the circulation of normal mice.. J Immunol.

[18] Choi JJ, Reich CF, Pisetsky DS (2005). The role of macrophages in the in vitro generation of extracellular DNA from apoptotic and necrotic cells.. Immunology.

[19] Pisetsky DS, Fairhurst AM (2007). The origin of extracellular DNA during the clearance of dead and dying cells.. Autoimmunity.

[20] Lo YM, Zhang J, Leung TN, Lau TK, Chang AM (1999). Rapid clearance of fetal DNA from maternal plasma.. Am J Hum Genet.

[21] Tukey RH, Strassburg CP (2000). Human UDP-glucuronosyltransferases: metabolism, expression, and disease.. Annu Rev Pharmacol Toxicol.

[22] Nakamura A, Nakajima M, Yamanaka H, Fujiwara R, Yokoi T (2008). Expression of UGT1A and UGT2B mRNA in human normal tissues and various cell lines.. Drug Metab Dispos.

[23] Hofmann T, Klenow S, Borowicki A, Gill CI, Pool-Zobel BL (2010). Gene expression profiles in human peripheral blood mononuclear cells as biomarkers for nutritional in vitro and in vivo investigations.. Genes Nutr.

[24] Johnson AD, Kavousi M, Smith AV, Chen MH, Dehghan A (2009). Genome-wide association meta-analysis for total serum bilirubin levels.. Hum Mol Genet.

[25] Lin JP, Schwaiger JP, Cupples LA, O'Donnell CJ, Zheng G (2009). Conditional linkage and genome-wide association studies identify UGT1A1 as a major gene for anti-atherogenic serum bilirubin levels–the Framingham Heart Study.. Atherosclerosis.

[26] Hong AL, Huo D, Kim HJ, Niu Q, Fackenthal DL (2007). UDP-Glucuronosyltransferase 1A1 gene polymorphisms and total bilirubin levels in an ethnically diverse cohort of women.. Drug Metab Dispos.

[27] Bosma PJ, Chowdhury JR, Bakker C, Gantla S, de Boer A (1995). The genetic basis of the reduced expression of bilirubin UDP-glucuronosyltransferase 1 in Gilbert's syndrome.. N Engl J Med.

[28] Botezatu I, Serdyuk O, Potapova G, Shelepov V, Alechina R (2000). Genetic analysis of DNA excreted in urine: a new approach for detecting specific genomic DNA sequences from cells dying in an organism.. Clin Chem.

[29] Ohta Y, Fukushima S, Yamashita N, Niimi T, Kubota T (2005). UDP-glucuronosyltransferase1A1 directly binds to albumin.. Hepatol Res.

[30] Nishimura Y, Maeda S, Ikushiro S, Mackenzie PI, Ishii Y (2007). Inhibitory effects of adenine nucleotides and related substances on UDP-glucuronosyltransferase: structure-effect relationships and evidence for an allosteric mechanism.. Biochim Biophys Acta.

[31] Barbier O, Turgeon D, Girard C, Green MD, Tephly TR (2000). 3′-azido-3′-deoxythimidine (AZT) is glucuronidated by human UDP-glucuronosyltransferase 2B7 (UGT2B7).. Drug Metab Dispos.

[32] Strassburg CP, Kneip S, Topp J, Obermayer-Straub P, Barut A (2000). Polymorphic gene regulation and interindividual variation of UDP-glucuronosyltransferase activity in human small intestine.. J Biol Chem.

[33] Lee TH, Montalvo L, Chrebtow V, Busch MP (2001). Quantitation of genomic DNA in plasma and serum samples: higher concentrations of genomic DNA found in serum than in plasma.. Transfusion.

[34] Umetani N, Hiramatsu S, Hoon DS (2006). Higher amount of free circulating DNA in serum than in plasma is not mainly caused by contaminated extraneous DNA during separation.. Ann N Y Acad Sci.

[35] Raitakari OT, Juonala M, Ronnemaa T, Keltikangas-Jarvinen L, Rasanen L (2008). Cohort profile: the cardiovascular risk in Young Finns Study.. Int J Epidemiol.

[36] Teo YY, Inouye M, Small KS, Gwilliam R, Deloukas P (2007). A genotype calling algorithm for the Illumina BeadArray platform.. Bioinformatics.

[37] Li Y, Willer C, Sanna S, Abecasis G (2009). Genotype imputation.. Annu Rev Genomics Hum Genet.

[38] Li Y, Willer CJ, Ding J, Scheet P, Abecasis GR (2010). MaCH: using sequence and genotype data to estimate haplotypes and unobserved genotypes.. Genet Epidemiol.

[39] Scuteri A, Sanna S, Chen WM, Uda M, Albai G (2007). Genome-wide association scan shows genetic variants in the FTO gene are associated with obesity-related traits.. PLoS Genet.

[40] Purcell S, Neale B, Todd-Brown K, Thomas L, Ferreira MA (2007). PLINK: a tool set for whole-genome association and population-based linkage analyses.. Am J Hum Genet.

[41] Aulchenko YS, Struchalin MV, van Duijn CM (2010). ProbABEL package for genome-wide association analysis of imputed data.. BMC Bioinformatics.

[42] Barrett JC, Fry B, Maller J, Daly MJ (2005). Haploview: analysis and visualization of LD and haplotype maps.. Bioinformatics.

